# Effects of adding a neurokinin-1 receptor antagonist to 5 mg olanzapine, a 5-hydroxytryptamine-3 receptor antagonist, and dexamethasone for preventing carboplatin-induced nausea and vomiting: a propensity score-matched analysis

**DOI:** 10.1186/s12885-022-09392-9

**Published:** 2022-03-23

**Authors:** Senri Yamamoto, Hirotoshi Iihara, Ryuji Uozumi, Hitoshi Kawazoe, Kazuki Tanaka, Yukiyoshi Fujita, Masakazu Abe, Hisao Imai, Masato Karayama, Yoh Hayasaki, Chiemi Hirose, Takafumi Suda, Kazuto Nakamura, Akio Suzuki, Yasushi Ohno, Ken-ichirou Morishige, Naoki Inui

**Affiliations:** 1 Department of Pharmacy, Gifu University Hospital, 1-1 Yanagido, Gifu, 501-1194 Japan; 2 Laboratory of Pharmacy Practice and Social Science, Gifu Pharmaceutical University, 1-25-4 Daigakunishi, Gifu, 501-1196 Japan; 3 Department of Biomedical Statistics and Bioinformatics, Kyoto University Graduate School of Medicine, 54 Kawahara-cho, Shogoin, Sakyo-ku, Kyoto, 606-8507 Japan; 4 Division of Pharmaceutical Care Sciences, Center for Social Pharmacy and Pharmaceutical Care Sciences, Keio University Faculty of Pharmacy, 1-5-30 Shibakoen, Minato-ku, Tokyo, 105-8512 Japan; 5 Division of Pharmaceutical Care Sciences, Keio University Graduate School of Pharmaceutical Sciences, 1-5-30 Shibakoen, Minato-ku, Tokyo, 105-8512 Japan; 6 Second Division, Department of Internal Medicine, Hamamatsu University School of Medicine, 1-20-1 Handayama, Hamamatsu, Shizuoka, 431-3192 Japan; 7 Division of Pharmacy, Gunma Prefectural Cancer Center, 617-1 Takahayashi-nishi, Ohta, Gunma 373-8550 Japan; 8 Division of Gynecology, Shizuoka Cancer Center, 1007 Shimonagakubo, Nagaizumi-cho, Sunto-gun, Shizuoka, 411-8777 Japan; 9 Department of Obstetrics and Gynecology, Hamamatsu University School of Medicine, 1-20-1 Handayama, Hamamatsu, Shizuoka, 431-3192 Japan; 10 Division of Respiratory Medicine, Gunma Prefectural Cancer Center, 617-1, Takahayashi-nishi, Ohta, Gunma 373-8550 Japan; 11 Department of Respiratory Medicine, Comprehensive Cancer Center, International Medical Center, Saitama Medical University, 1397-1 Yamane, Hidaka, Saitama 350-1298 Japan; 12 Department of Clinical Oncology, Hamamatsu University School of Medicine, 1-20-1 Handayama, Hamamatsu, Shizuoka 431-3192 Japan; 13 Department of Obstetrics and Gynecology, Gifu University Graduate School of Medicine, 1-1 Yanagido, Gifu, 501-1194 Japan; 14 Department of Gynecology, Gunma Prefectural Cancer Center, 617-1 Takahayashi-nishi, Ohta, Gunma 373-8550 Japan; 15 Department of Cardiology and Respiratory Medicine, Gifu University Graduate School of Medicine, 1-1 Yanagido, Gifu, 501-1194 Japan; 16 Department of Clinical Pharmacology and Therapeutics, Hamamatsu University School of Medicine, 1-20-1 Handayama, Hamamatsu, Shizuoka 431-3192 Japan

**Keywords:** Antiemetics, Carboplatin, Dexamethasone, Nausea, Neurokinin-1 receptor antagonist, Olanzapine, Vomiting, 5-hydroxytryptamine-3 receptor antagonists

## Abstract

**Background:**

Olanzapine has been reported to be an effective antiemetic in patients receiving carboplatin-based chemotherapy. However, the efficacy of a neurokinin-1 receptor antagonist (NK_1_RA) added to olanzapine, a 5-hydroxytryptamine-3 receptor antagonist (5-HT_3_RA), and dexamethasone (DEX) has not been proven. This study aimed to assess the efficacy and safety of NK_1_RA, in combination with three-drug antiemetic regimens containing olanzapine, in preventing nausea and vomiting induced by carboplatin-based chemotherapy.

**Methods:**

Data were pooled for 140 patients receiving carboplatin-based chemotherapy from three multicenter, prospective, single-arm, open-label phase II studies that evaluated the efficacy and safety of olanzapine for chemotherapy-induced nausea and vomiting. The propensity score of the co-administration of NK_1_RA was estimated for each patient using a logistic regression model that included age, sex, and carboplatin dose. We analyzed a total of 62 patients, who were treated without NK_1_RA (non-NK_1_RA group: 31 patients) and with NK_1_RA (NK_1_RA group: 31 patients). The patients were selected using propensity score matching.

**Results:**

The complete response rate (without emetic episodes or with no administration of rescue medication) in the overall period (0–120 h post carboplatin administration) was 93.5% in the non-NK_1_RA group and 96.8% in the NK_1_RA group, with a difference of -3.2% (95% confidence interval, -18.7% to 10.9%; *P* = 1.000). In terms of safety, there was no significant difference between the groups in daytime sleepiness and concentration impairment, which are the most worrisome adverse events induced by olanzapine.

**Conclusions:**

The findings suggest that antiemetic regimens consisting of olanzapine, 5HT_3_RA, and DEX without NK_1_RA may be a treatment option for patients receiving carboplatin-based chemotherapy.

## Background

Carboplatin is classified as a moderate-emetic-risk chemotherapy (MEC) or high-emetic-risk chemotherapy (HEC) [1–4]. Jordan et al. conducted a systematic review and meta-analysis of randomized controlled trials that assessed the effects of adding a neurokinin-1 receptor antagonist (NK_1_RA) to a 5-hydroxytryptamine-3 receptor antagonist (5-HT_3_RA) and dexamethasone (DEX) in MEC [5]. In this study, a total of 1790 patients from seven trials were analyzed, and the results of 1538 patients for whom complete response (CR) rate could be assessed supported the NK_1_RA combined regimen for carboplatin-based chemotherapy with an absolute risk difference of 15% and an odds ratio of 1.96 (95% confidence interval [CI]: 1.57–2.45; *p* < 0.001). Currently, international antiemetic guidelines consistently recommend a three-drug antiemetic prophylaxis with NK_1_RA, 5-HT_3_RA, and DEX in patients receiving carboplatin-based chemotherapy [1–4].

Olanzapine is an antipsychotic drug that is classified as a multi-acting, receptor-targeted agent. It has been reported to be a highly effective antiemetic drug in patients receiving MEC and/or HEC [6–12]. Three high-quality phase II studies have reported the efficacy and safety of 5 mg olanzapine for antiemetic prophylaxis in patients receiving carboplatin-based chemotherapy [13–15]. Two of these studies evaluated the antiemetic effects of a four-drug combination consisting of olanzapine, NK_1_RA, 5-HT_3_RA, and DEX, and one evaluated a three-drug combination consisting of olanzapine, 5-HT_3_RA, and DEX. To the best of our knowledge, there are no phase III studies evaluating the efficacy and safety of olanzapine for the management of nausea and vomiting in cancer patients receiving carboplatin-based chemotherapy. Therefore, we integrated these three phase II studies and reported the efficacy and safety of olanzapine in patients receiving carboplatin-based chemotherapy and the risk factors associated with carboplatin-induced nausea and vomiting [16].

The results showed that olanzapine had an antiemetic effect with a CR rate (defined as no emetic episodes and no administration of rescue medication for nausea and vomiting) of 87.9% in the overall period (0–120 h). In the analysis of risk factors affecting carboplatin-induced nausea and vomiting, co-administration of NK_1_RA was not significantly associated with carboplatin-induced nausea and vomiting. This integrated analysis is the only study that analyzes the effect of NK_1_RA, when added to an olanzapine-containing antiemetic regimen, on carboplatin-induced nausea and vomiting. However, the efficacy of NK_1_RA in combination with an olanzapine-containing antiemetic regimen remains to be demonstrated. Therefore, the present study aimed to evaluate the efficacy and safety of the combination of NK_1_RA, olanzapine, 5-HT_3_RA, and DEX in preventing carboplatin-induced nausea and vomiting in a propensity score-matched analysis.

## Methods

### Study design

We analyzed 62 patients, treated without NK_1_RA (non-NK_1_RA group, 31 patients) and with NK_1_RA (NK_1_RA group: 31 patients), using a propensity score-matched sample from the pooled data of 140 patients receiving carboplatin-based chemotherapy. The data were from three multicenter, prospective, single-arm, open-label, phase II studies.

**Fig. 1:**
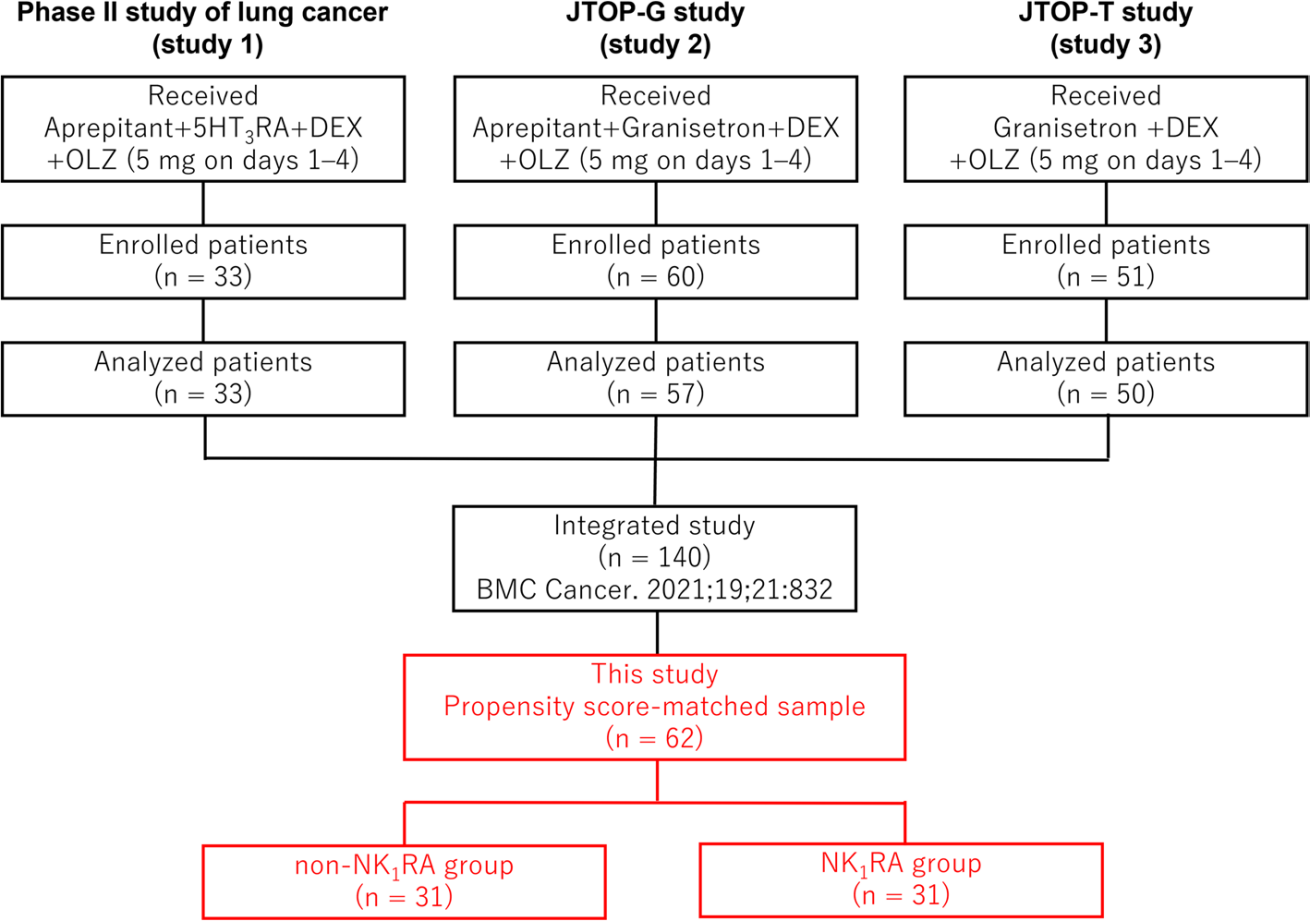
Patient enrollment flowchart. In all, 62 patients were selected using the propensity score-matched sample from three multicenter, prospective, single-arm, open-label, phase II studies. 5-HT_3_RA, 5-hydroxytryptamine-3 receptor antagonists; DEX, dexamethasone; OLZ, olanzapine

The results of these three phase II studies and the integrated analysis of the pooled data of 140 patients have been published previously [13–16]. Study 1 reported the efficacy of a four-drug combination consisting of olanzapine (orally: 5 mg on days 1–4), aprepitant (orally: 125 mg on day 1 and 80 mg on days 2 and 3), 5-HT_3_RA (intravenously: granisetron 1 mg, granisetron 3 mg, palonosetron 0.75 mg, or ramosetron 0.3 mg on day 1), and DEX (intravenously: 4.95 mg on day 1) in 33 patients with lung cancer [13]. Study 2 reported the efficacy of a four-drug combination consisting of olanzapine (orally: 5 mg on day 1 to 4), aprepitant (orally: 125 mg on day 1 and 80 mg on days 2 and 3), granisetron (intravenously: 1 mg on day 1), and DEX (intravenously: 9.9 mg on day 1) in 57 patients with gynecological cancer [14]. Study 3 reported the efficacy of a three-drug combination consisting of olanzapine (orally: 5 mg on day 1 to 4), granisetron (intravenously: 1 mg on day 1), and DEX (intravenously/orally: 9.9 mg/12 mg on day 1 and 6.6 mg/8 mg on days 2 and 3) in 50 patients with thoracic malignancies [15]. The patient enrollment flowchart for the present study is shown in Fig. 1.

### Data collection

Data were collected from self-reported diaries. Patients reported nausea, decreased appetite, somnolence, and decreased concentration severity using a four-point scale (none, mild, moderate, and severe), as well as frequency of vomiting, and the use of rescue medication. The daily diary began from the initiation of carboplatin treatment on day 1, and entries were made over a 5-day period (Studies 1 and 3) and a 7-day period (Study 2).

### Outcome

The primary endpoints for efficacy were CR rate, defined as the proportion of patients without emetic episodes or administration of rescue medication; complete control (CC) rate, defined as the proportion of patients with CR and no more than mild nausea; and total control (TC) rate, defined as the proportion of patients with CR and no nausea. The assessment periods for carboplatin-induced nausea and vomiting were 0–120 h post carboplatin administration (overall period), 0–24 h post carboplatin administration (acute period), and 24–120 h post carboplatin administration (delayed period). Additionally, the secondary endpoints for efficacy were incidences of nausea, vomiting, and decreased appetite for 5 days after the initiation of carboplatin treatment on day 1.

The endpoints for safety were incidences of somnolence and decreased concentration for 5 days after the initiation of carboplatin treatment on day 1.

### Statistical analysis

Patient characteristics, rate of carboplatin-induced nausea and vomiting control, and treatment-related adverse events were summarized using descriptive statistics or reported in terms of frequencies and proportions of total patients. The propensity score of the co-administration of NK_1_RA was estimated for each patient using a logistic regression model that included age, sex, and carboplatin dose which most potentially affect the occurrence of chemotherapy-induced nausea and vomiting (CINV) in patients [17–20]. In the propensity score matching, 1:1 nearest neighbor matching algorithm without replacement was employed with a caliper width equal to 0.2 of the standard deviation of the logit of the propensity score [21]. The difference in the primary endpoints between the NK_1_RA and non-NK_1_RA groups was shown with a two-sided exact CI [22] and compared using Fisher’s exact test. All statistical analyses were performed using JMP 15.0.0 and SAS version 9.4 (SAS Institute, Inc., Cary, NC, USA). All *P*-values were two-sided, and statistical significance was set at *P* < 0.05.

## Results

### Study patients

A total of 62 patients were included in the analysis. Of these patients, 31 were in the non-NK_1_RA group and 31 in the NK_1_RA group. Baseline patient characteristics are presented in Table 1. The median ages of patients in the non-NK_1_RA group and those in the NK_1_RA group were 71 years (range, 25th and 75th percentiles, 67–76 years) and 71 years (range, 25th and 75th percentiles, 65–77 years), respectively. The proportion of males (58.1%) to females (41.9%) was similar in both groups.

**Table 1:** Baseline patient characteristics

	Non-NK_1_RA group	NK_1_RA group
	(*n* = 31)	(*n* = 31)
Age, years		
Median (interquartile range)	71 (67–76)	71 (65–77)
< 60 years	2 (6.5%)	2 (6.5%)
≥ 60 years	29 (93.5%)	29 (93.5%)
Sex		
Male	18 (58.1%)	18 (58.1%)
Female	13 (41.9%)	13 (41.9%)
ECOG performance status		
0	14 (45.2%)	28 (90.3%)
1	12 (38.7%)	2 (6.5%)
2	5 (16.1%)	1 (3.2%)
Cancer type		
Small-cell lung cancer	9 (29.0%)	8 (25.8%)
Non-small-cell lung cancer	17 (54.8%)	14 (45.2%)
Thymoma / thymic carcinoma	5 (16.1%)	0 (0.0%)
Ovarian cancer	0 (0.0%)	4 (12.9%)
Endometrial cancer	0 (0.0%)	4 (12.9%)
Peritoneal cancer	0 (0.0%)	1 (3.2%)
Planned carboplatin dose		
AUC 5 mg/mL/min	21 (67.7%)	21 (67.7%)
AUC 6 mg/mL/min	10 (32.3%)	10 (32.3%)
Additional anticancer drugs		
Paclitaxel	3 (9.7%)	9 (29.0%)
Paclitaxel + Pembrolizumab	1 (3.2%)	0 (0.0%)
Paclitaxel + Bevacizumab	0 (0.0%)	1 (3.2%)
Paclitaxel + Bevacizumab + Atezolizumab	2 (6.5%)	0 (0.0%)
Nab-Paclitaxel	0 (0.0%)	3 (9.7%)
Nab-Paclitaxel + Pembrolizumab	3 (9.7%)	0 (0.0%)
Pemetrexed	7 (22.6%)	6 (19.4%)
Pemetrexed + Pembrolizumab	2 (6.5%)	0 (0.0%)
Pemetrexed + Bevacizumab	0 (0.0%)	2 (6.5%)
Etoposide	8 (25.8%)	8 (25.8%)
Etoposide + Atezolizumab	2 (6.5%)	0 (0.0%)
Vinorelbine	2 (6.5%)	0 (0.0%)
S-1	1 (3.2%)	2 (6.5%)
Risk factor		
Habitual alcohol consumption	19 (61.3%)	10 (32.3%)
Motion sickness	25 (80.6%)	2 (6.5%)
Morning sickness	3 (9.7%)	7 (22.6%)

Data are n (%)

*ECOG* Eastern Cooperative Oncology Group

*AUC* Area under the curve

*S-1* tegafur plus gimeracil plus oteracil potassium

### Efficacy

The primary endpoints of efficacy are shown in Table 2. As shown in the table, CR rates for the overall, delayed, and acute periods in the non-NK_1_RA and NK_1_RA groups did not show any statistically significant difference. Likewise, the CC and TC rates in the non-NK_1_RA group, during each period, were not significantly different from those in the NK_1_RA group.

**Table 2:** Primary endpoint for efficacy

Outcome	Non-NK_1_RA group	NK_1_RA group	Risk Difference	*P* value
	(*n* = 31)	(*n* = 31)	(95% CI)	
CR				
Overall	29 (93.5%)	30 (96.8%)	-3.2% (-18.7 to 10.9)	1.000
Acute	31 (100%)	31 (100%)	0%	
Delayed	29 (93.5%)	30 (96.8%)	-3.2% (-18.7 to 10.9)	1.000
CC				
Overall	29 (93.5%)	29 (93.5%)	0% (-16.6 to 16.6)	1.000
Acute	31 (100%)	31 (100%)	0%	
Delayed	29 (93.5%)	29 (93.5%)	0% (-16.6 to 16.6)	1.000
TC				
Overall	27 (87.1%)	26 (83.9%)	3.2% (-16.6 to 22.9)	1.000
Acute	31 (100%)	30 (96.8%)	3.2% (-8.4 to 16.7)	1.000
Delayed	27 (87.1%)	27 (87.1%)	0% (-19.0 to 19.0)	1.000

*CR* Complete response, *CC* Complete control, *TC* Total control, *CI* Confidence interval

**Fig. 2:**
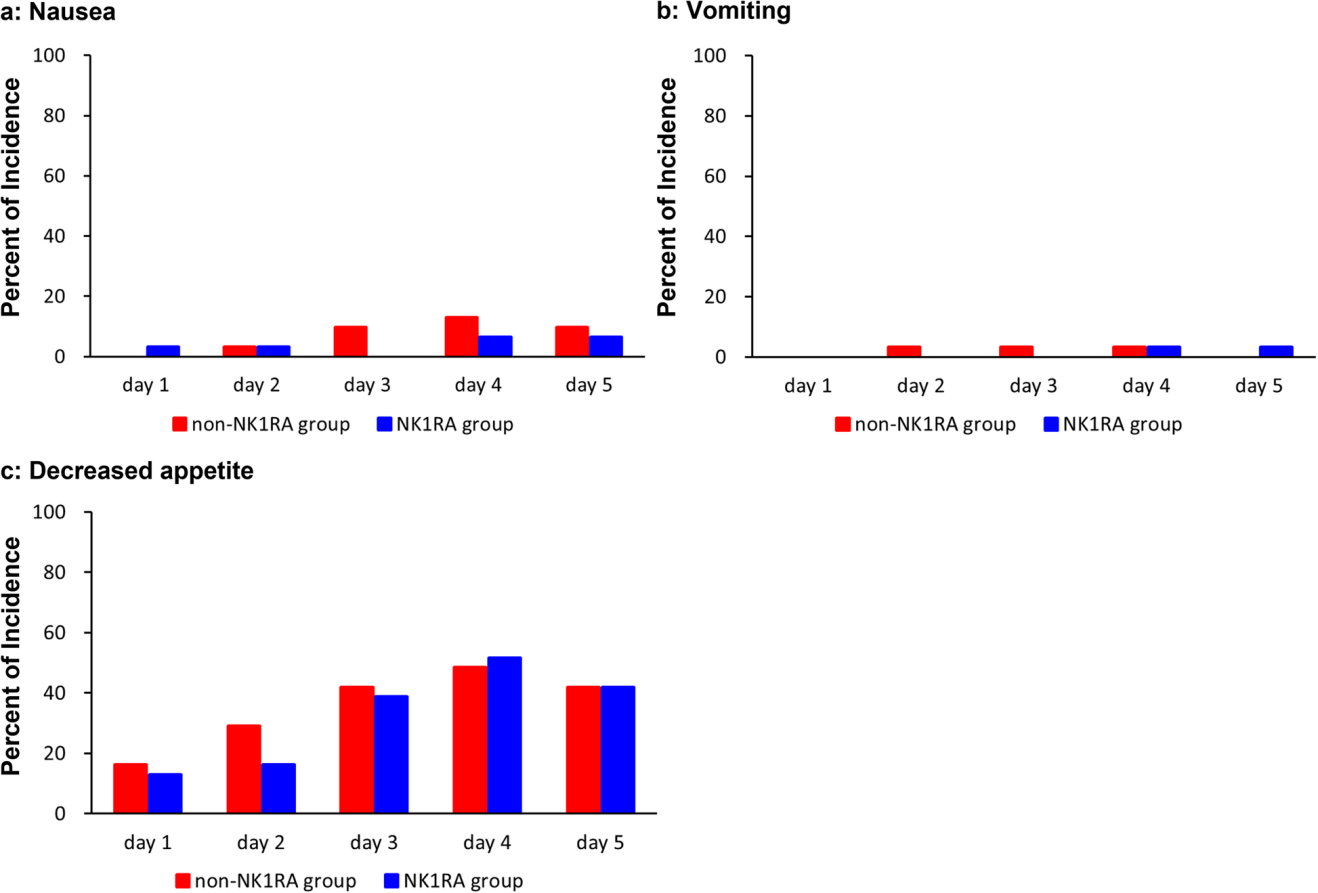
Incidences of nausea, vomiting and decreased appetite for 5 days after the initiation of carboplatin treatment on day 1

The secondary endpoints for efficacy are shown in Fig. 2. Patient-reported nausea, vomiting, and decreased appetite in the overall period were not significantly different between the two groups. The incidence of nausea was 12.9% in the non-NK_1_RA group and 16.1% in the NK_1_RA group (*P* = 1.000), that of vomiting was 6.5% in the non-NK_1_RA group and 3.2% in the NK_1_RA group (*P* = 1.000), and that of decreased appetite was 58.1% in the non-NK_1_RA group and 61.3% in the NK_1_RA group (*P* = 1.000).

### Safety

**Fig. 3:**
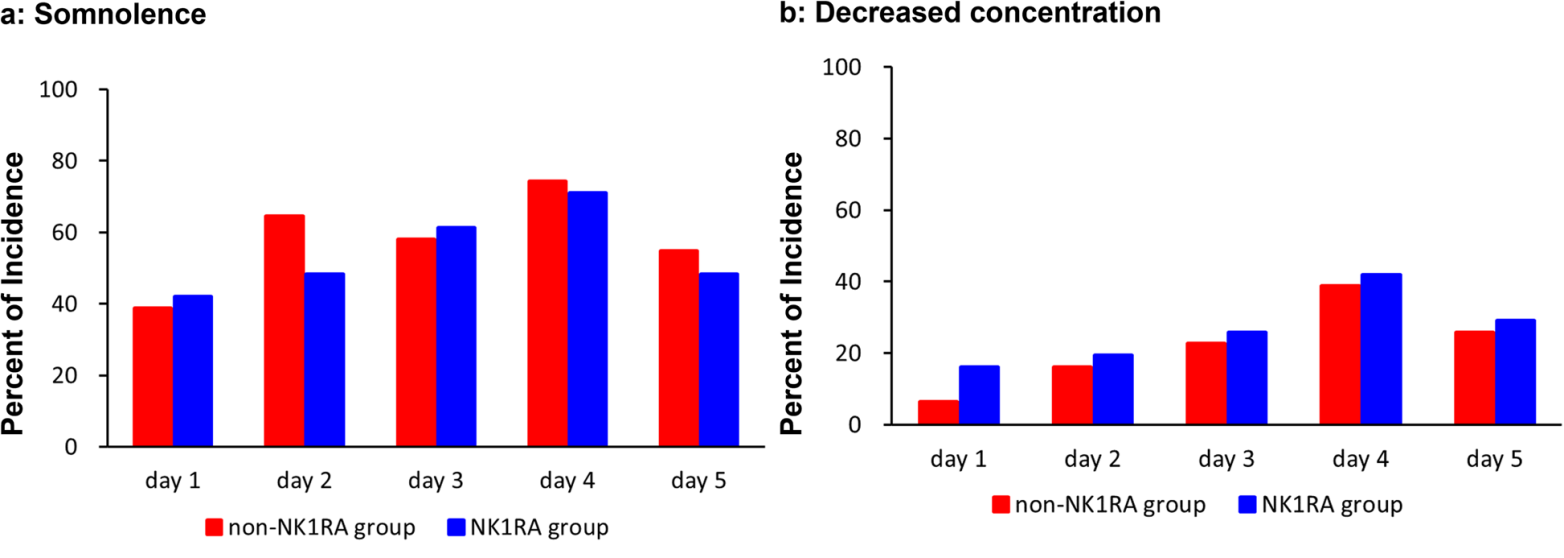
Incidences of somnolence and decreased concentration for 5 days after the initiation of carboplatin treatment on day 1

Data on somnolence and decreased concentration assessed by the patients’ self-reported diaries are shown in Fig. 3. The incidence of somnolence was 83.9% in the non-NK_1_RA group and 80.6% in the NK_1_RA group. However, moderate or severe somnolence was 6.5% in the non-NK_1_RA group and 0% in the NK_1_RA group. The incidence of decreased concentration was 48.4% in the non-NK_1_RA group and 48.4% in the NK_1_RA group. However, moderate or severe decreased concentration was 3.2% in the non-NK_1_RA group and 0% in the NK_1_RA group. The peak incidence of somnolence and decreased concentration was observed on day 4 in both groups.

## Discussion

To the best of our knowledge, there are no studies that have evaluated the efficacy of adding NK_1_RA to antiemetic therapy consisting of olanzapine, 5HT_3_RA, and DEX in MEC and HEC. In the present study, the prophylactic antiemetic combination regimen of olanzapine, 5-HT_3_RA, and DEX showed no statistical difference between groups treated with or without NK_1_RA for CINV control, as demonstrated by the endpoints of CR, CC, and TC rates during the overall, acute, and delayed periods. Moreover, daytime sleepiness and concentration impairment, the most worrisome adverse events associated with olanzapine administration, were unaffected by NK_1_RA administration. The incidences of moderate and severe daytime sleepiness and concentration impairment were rare.

In the present study, prophylactic antiemetic treatment without NK_1_RA had a high CR rate of 93.5%, CC rate of 93.5%, and TC rate of 87.1%. The incidence of nausea in the non-NK_1_RA group was also very low (12.9%). A head-to-head comparison of the antiemetic effects of olanzapine and NK_1_RA, each combined with palonosetron and DEX, has been reported for patients receiving HEC. [6, 7]. In these studies, the CR rates of the olanzapine and NK_1_RA regimens were comparable in the overall, acute, and delayed periods. Nevertheless, antiemetic prophylaxis with the olanzapine regimen resulted in a significantly higher control of nausea in the delayed and overall periods than that with the NK_1_RA regimen. It has been reported that when 5-HT released by anticancer drugs acts on 5-HT_2b_ and 5-HT_2c_ receptors, the secretion of ghrelin, an appetite-stimulating hormone, is decreased, inducing anorexia and nausea [23]. Olanzapine is an antipsychotic drug classified as a multi-acting, receptor-targeted agent that is known to antagonize 5-HT at the 5-HT_2b_ and 5-HT_2c_ receptors [24]. These mechanisms may account for the excellent nausea-suppressing effects of olanzapine.

The incidence of nausea, vomiting, and decreased appetite mainly peaked on day 4 in both groups, which is consistent with a recent report by Iihara et al. showing that CINV associated with carboplatin occurs on day 4 [25].

Younger age is a well-known patient-related risk factor for CINV [17–20]. In our previous study, which evaluated the efficacy of olanzapine for carboplatin-induced nausea and vomiting in younger patients, the cut-off value for age was set to 60 years, and was significantly associated with an increased risk of non-TC in the overall study period [16]. The median patient age in the present study was 71 years (range, 25th and 75th percentiles, 67–76 years) in the non-NK_1_RA group and 71 years (range, 25th and 75th percentiles, 65–77 years) in the NK_1_RA group, which had relatively older patients. Only two patients under the age of 60 years were included in both groups. Therefore, caution should be exercised when extrapolating the results of this study to younger patients, especially those aged below 60 years. We suggest that these findings should be confirmed with a randomized comparison of older and younger patients in future research.

Undesired patient sedation with 10 mg olanzapine is a problem in its antiemetic use for elderly or oversedated patients [1, 3, 11]. The J-FORCE study, which evaluated 5 mg olanzapine in patients receiving high-dose cisplatin, suggested that 5 mg olanzapine therapy does not have a significant effect on daytime somnolence and decreased concentration [12]. Our previously reported integrated analysis evaluating 5 mg of olanzapine in patients receiving carboplatin was consistent with this result [16]. This was not affected by the presence or absence of the NK_1_RA combination.

The present study has some limitations. First, this study had an open-label, single-arm design. Second, data was small number from three studies. But we used a propensity score-matched analysis which is a popular methodology for a retrospective study design. Third, the results of this study are not a direct comparison between patients treated with or without NK_1_RA. Furthermore, due to the older age of the patients included in this analysis, the results may not be applicable to younger patients. Finally, the results were obtained only in the Japanese population. In the future, a phase III trial comprising a direct comparison of the efficacy and safety of an antiemetic combination regimen of olanzapine, 5-HT_3_RA, and DEX, with or without NK_1_RA in patients receiving carboplatin-based chemotherapy is warranted.

## Conclusion

These findings suggest that antiemetic combination regimens of olanzapine, 5-HT_3_RA, and DEX without NK_1_RA may be a treatment option for patients treated with carboplatin-based combination chemotherapy with an area under the curve of ≥ 5 mg/mL/min.

## Data Availability

The data that support the findings of this study are available from the study groups, but restrictions apply to the availability of these data, which were used under license for the current study; therefore, the data are not publicly available. However, data are available from the corresponding authors upon reasonable request and with permission from the study groups.

## Abbreviations

5-HT_3_RA: 5-Hydroxytryptamine-3 receptor antagonist
CC: Complete control
CI: Confidence interval
CINV: Chemotherapy-induced nausea and vomiting
CR: Complete response
DEX: Dexamethasone
ECOG PS: Eastern Cooperative Oncology Group performance status
HEC: High-emetic-risk chemotherapy
MEC: Moderate-emetic-risk chemotherapy
NK_1_RA: Neurokinin-1 receptor antagonist
TC: Total control
